# Burosumab prevents further height deficit in toddlers affected by XLH

**DOI:** 10.1530/EC-25-0435

**Published:** 2025-10-18

**Authors:** Elisa Sala, Jugurtha Berkenou, Anya Rothenbuhler, Anne-Sophie Lambert, Christelle Audrain, Barbara Girerd, Marco Pitea, Stefano Mora, Agnès Linglart, Diana-Alexandra Ertl

**Affiliations:** ^1^ AP HP, Department of Endocrinology and Diabetology for Children and Department of Adolescent Medicine, Bicêtre Paris-Saclay University Hospital, Le Kremlin Bicêtre, France; ^2^ AP HP, Reference Center for Rare Disorders of The Calcium and Phosphate Metabolism, Filière OSCAR and Platform of Expertise for Rare Diseases Paris-Saclay, EndoRare and BOND ERN, Bicêtre Paris Saclay Hospital, Le Kremlin-Bicêtre, France; ^3^ Department of Endocrinology and Diabetology for Children, San Raffaele Hospital, Milan, Italy; ^4^ University “Vita-Salute San Raffaele”, Milan, Italy; ^5^ Université Paris-Saclay, Inserm, Physiologie et Physiopathologie Endocriniennes, Le Kremlin-Bicêtre, France

**Keywords:** XLH, height, PHEX, short stature, young pediatric population

## Abstract

**Background:**

X-linked hypophosphatemia (XLH) is a rare disease caused by *PHEX* variants. Besides rickets, XLH leads to disproportionately short stature, which develops during the first months of life. Burosumab afforded minimal improvement of growth in children above the age of 4 years. No data are available on growth, including body mass index, of XLH children who started burosumab at a very young age, i.e., between 1 and 4 years.

**Methods:**

We performed a prospective follow-up of growth and other XLH-related outcomes in XLH children who started burosumab before the age of 4 years. We compared these children 1:2 with a historical cohort of XLH children who started vitamin D analogs and phosphate supplements before the age of 4 years.

**Results:**

We included 15 children treated with burosumab and 31 children treated with vitamin D analogs and phosphate supplements. In the burosumab-treated group, mean ± SD for age at therapy baseline was 2.1 ± 0.7 years (range: 1–2.9 years). They were treated with oral phosphate and active vitamin D for 1.7 ± 0.8 years before switching to burosumab. From birth to burosumab start, they presented a decline in height standard deviation score (SDS) from −0.3 ± 0.7 to −1.4 ± 0.8 (mean ± SD), respectively, *P* < 0.001. On burosumab, height SDS did not decline further during the first 2 years of treatment: mean ± SD 0.1 ± 0.6 (range: −0.7–1.3 SDS) after 1 year (*P* = 0.16) and 0.0 ± 0.7 SD (range: −0.6–1.4 SDS) after 2 years (*P* = 0.54). Burosumab did not correct the acquired height deficit, as children had a difference in height SDS of −1.5 SDS after 2 years of therapy when compared to birth length SDS (*P* = 0.04). BMI SDS did not significantly change during the first 2 years on burosumab. Children treated with vitamin D analogs and phosphate supplements started treatment at a mean ± SD age of 1.3 ± 0.7 years (range: 0.1–3.0 years) and presented a continuous decline in height SDS of 0.7 ± 0.9 SDS (range: −2.6–1) during the first 2 years of therapy (*P* < 0.001) and up to 4 years of age (−1.8 ± 0.9 SDS to −1.9 ± 0.9, respectively). BMI SDS increased by 0.5 ± 0.9 SDS (range: −0.6–1.9) during the same period (*P* = 0.006).

**Conclusion:**

We present data from the largest pediatric XLH cohort of very young children treated with burosumab over a follow-up period of 2 years. Our data suggest that, in contrast to the combination of vitamin D analogs and phosphate supplements, burosumab prevents further height deficit in XLH children, even at a period of life associated with high growth velocity. In addition, burosumab prevents the early and excessive weight gain associated with the development of XLH in children.

**Significance statement:**

Most patients with X-linked hypophosphatemic rickets (XLH) present with short stature. This is the first large study on XLH children younger than 5 years treated with burosumab since a mean age of 2.1 years. We analyzed growth over a period of 2 years of therapy. Our data show that, in XLH toddlers, burosumab therapy prevents the further loss of height and the weight gain observed in patients treated with oral phosphate and active vitamin D therapy at the same age. As burosumab leads to better long-term outcomes in these children, we hope that our work will encourage the early use of burosumab and motivate further research in this field.

## Background

X-linked hypophosphatemia (XLH) is the most common cause of genetic rickets, with a prevalence of approximately 1 in 20,000–60,000 people worldwide. It is caused by loss-of-function variants in the phosphate-regulating endopeptidase homolog on the X chromosome (*PHEX*) gene. *PHEX* is located on Xp22.11 and encodes a membrane protein expressed in bone and tooth tissues. Pathogenic variants of *PHEX* cause increased levels of fibroblast growth factor 23 (FGF23) (1, 2).

FGF23, along with parathyroid hormone (PTH) and calcitriol (1,25-dihydroxyvitamin D), is a main regulatory hormone of phosphate homeostasis (3, 4). In XLH, excessive FGF23 production leads to decreased renal phosphate reabsorption and, as a result, chronic hypophosphatemia (5). Lower limb deformities, together with short stature, are the most frequent and severe complications of XLH during the first years of life. They motivate early diagnosis and treatment of the disease (6).

Since the 1980s, children and adults with XLH have been treated with the combination of multiple daily oral doses of phosphate and active vitamin D analogs. Although this therapy has brought about significant clinical and biochemical improvements, its effect on growth has remained moderate (1, 7, 8).

Burosumab, a human monoclonal anti-FGF23 antibody, has been approved since 2018 in Europe, the United States, and Canada for the treatment of XLH. Burosumab blocks the action of FGF23 and restores the renal reabsorption of phosphate and the expression of 1-alpha hydroxylase, leading thereby to an increase in plasma levels of phosphate and 1,25-dihydroxyvitamin D. It therefore significantly and rapidly improves the signs of rickets (1, 9, 10).

Impaired growth is a hallmark of XLH. Although observational studies have shown increased growth rate with phosphate supplements and active vitamin D analogs, the effect is moderate, and 40–50% of patients with well-controlled rickets still present with short stature, i.e., adult height below −2 SDSs (standard deviation scores), despite optimal treatment (11). Furthermore, the blockade of FGF23 signaling has a very limited effect on growth velocity, as shown in phase 2 and phase 3 clinical trials, and reported from real-life observations (12, 13, 14, 15). Only two of these clinical trials presented data on growth under burosumab in children younger than the age of five (12, 13).

Over the years, recombinant human growth hormone (rhGH) therapy has been used in XLH patients and has led to improvement in longitudinal growth without aggravating body disproportion, both in patients treated with phosphate supplements and vitamin D analogs, and in patients treated with burosumab. However, more clinical studies are needed to evaluate the long-term effects on adult height of rhGH in combination with burosumab (16).

Therefore, despite the development of new therapeutic approaches, height deficit remains a major feature of XLH. We therefore decided to explore the growth of very young XLH children treated with phosphate supplements and vitamin D analogs, and of patients treated with burosumab.

## Study objectives

We compared linear growth (Δ height SDS) between children who started burosumab before 4 years of age and children treated with phosphate supplements and vitamin D analogs before the age of 4 years.

We also studied the change in body mass index in SDSs (ΔBMI SDS) in both groups.

The impact of treatment regimens on rickets was measured through alkaline phosphatase levels at diagnosis and at specific time points. In addition, we compared serum alkaline phosphatase levels between the two groups.

## Materials and methods

### Study design

We included in the current study all children with XLH, aged 1 to 5 years, born between 2001 and 2021, who underwent regular follow-up at our hospital and had accurate auxological and biochemical data available.

Since March 1st, 2018, all XLH children treated with burosumab have been included in a prospective longitudinal single-center cohort at the French National Reference Center for Rare Disorders of Calcium and Phosphate Metabolism, Filière OSCAR, and Platform of expertise for rare disease, ENDO-ERN and ERN-BOND, Bicêtre Paris-Saclay Hospital, France. The cohort study is registered with ClinicalTrials.gov (identifier NCT04419363; https://clinicaltrials.gov/study/NCT04419363).

The diagnosis of XLH was based on clinical, biochemical, and radiological features and confirmed by genetic analysis. In some children, the diagnosis was made early in life based on positive family history.

We collected clinical and biochemical data from patients’ medical records, including genetic and therapeutic information. We then divided the XLH study population into two groups based on their treatment regimen, i.e., vitamin D analogs and phosphate supplements, or burosumab. Children treated with phosphate supplements and vitamin D analogs constitute a historical control group. Children additionally treated with recombinant growth hormone (rhGH) for short stature were not included in the study. All patients treated with burosumab initially received vitamin D analogs and phosphate supplements.

Phosphate and active vitamin D supplementation were prescribed according to international recommendations, i.e., starting dose of elemental phosphorus of 20–60 mg/kg body weight daily and calcitriol initial dose of 20–30 ng/kg body weight daily, or alfacalcidol of 30–50 ng/kg body weight daily (1).

Burosumab was started in accordance with international guidelines at the dose of 0.4 mg/kg body weight or 0.8 mg/kg body weight administered subcutaneously every 2 weeks, after discontinuing oral phosphate and active vitamin D therapy for at least 1 week before starting burosumab. Dose increments up to a maximum dose of 2 mg/kg were performed by assessing the combination of serum phosphate and alkaline phosphatase levels, and patient clinical improvements (1, 17).

### Study dataset

Data were collected at the time of diagnosis, at the beginning of the treatment (burosumab or vitamin D analogs and phosphate supplements), and every 6 months for the first 2 years of therapy. In addition, for both groups, we evaluated auxological and biochemical data at 4 years of age. Familial data (family history of XLH, parents’ height), birth data (gestational week, birth weight, length, and head circumference), as well as genetic data, were collected.

We collected auxological data, such as height (cm) and BMI (kg/m^2^). For the measurement of height, we used a Harpenden stadiometer. These parameters, as well as calculated BMI, were analyzed as SDS, using WHO reference data for height and BMI (https://www.who.int/tools/child-growth-standards/software).

We calculated the change in height and BMI in SDS (Δ SDS) between baseline (start of therapy, phosphate supplements and vitamin D analogs, or burosumab) and 12 and 24 months of therapy.

Subjects were excluded from the study if they had other health problems that could have adversely affected growth (e.g., inflammatory bowel diseases, chronic systemic diseases).

Data on parental height and genetic target height were not included in this analysis because of the high prevalence of familial XLH cases in the study sample, which could affect the interpretation.

We also collected biochemical parameters that are analyzed in the routine follow-up of XLH, such as serum concentrations of phosphate, calcium, parathyroid hormone (PTH), alkaline phosphatase (ALP), 25-hydroxyvitamin D, 1,25-dihydroxyvitamin D, creatinine, as well as urinary levels of calcium, phosphate, and creatinine for calculation of the tubular maximum reabsorption of phosphate per glomerular filtration rate (TmP/GFR), and urinary calcium/creatinine ratio. We only present these data in a descriptive manner (see Supplemental Table (see section on Supplementary materials given at the end of the article)).

### Statistical analysis

The descriptive analysis of the study sample was performed using IBM SPSS Statistics 25.0 software. Continuous variables with a normal distribution are described using the mean, SD, and range (minimum and maximum). Height in centimeters and body mass index in kilograms per square meter of height were plotted on WHO growth curves for each participant in both groups.

For the comparison of ΔH SDS between the two groups and within the groups, we used an analysis of covariance model (PROC GLM in SAS 9.4). Mean differences were estimated from this model with 95% confidence intervals. *P*-values below 0.05 are considered to indicate statistical significance. The one-way ANOVA test, followed by Tukey’s multiple comparisons test was used to compare the differences between the calculated means for the analyzed parameters over the study period. We used the unpaired *t*-test to compare data between the two study groups.

## Results

### Characteristics of the study population

**Figure 1:**
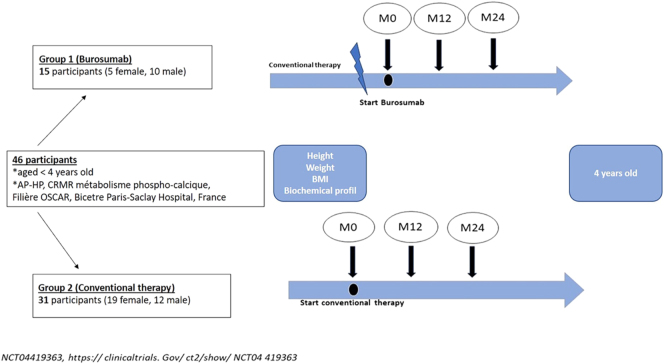
Study design and groups. M0: initiation of therapy, M12: 12 months after treatment initiation, M24: 24 months after treatment initiation. CT: conventional therapy (i.e., vitamin D analogs and phosphate supplements).

We included a total of 46 XLH children, as illustrated in Fig. 1 and Table 1.

**Table 1:** Characteristics and biochemical parameters of our XLH population.

Parameters	Group 1 (burosumab)		Group 2 (phosphate supplements and vitamin D analogs)	
	*n* (%)		*n* (%)	
Gender	Male	10 (66.6%)	Male	12 (38.7%)
	Female	5 (33.3%)	Female	19 (61.3%)
Family history	Positive	14 (93.4%)	Positive	25 (80.6%)
	Absent	1 (6.6%)	Absent	6 (19.4%)

	Mean ± SD	Min/max	Mean ± SD	Min/max
Age at diagnosis (years)	0.49 ± 0.8	0.0–2.4	1.2 ± 0.7	0.1–3
Phosphate levels at diagnosis (mmol/L)	1.14 ± 0.25	0.81/1.5	0.84 ± 0.22	0.53/1.3
ALP levels at diagnosis (IU/L)	744 ± 226	460/1,072	885 ± 617	365/2,432
Age at start of phosphate supplements and vitamin D analogs (years)	0.5 ± 0.8	0.1–2.5	1.3 ± 0.7	0.1–3.0

Mean ± SD for age at diagnosis, age at the start of conventional therapy, and at the start of burosumab. Mean ± SD, minimum, and maximum for biochemical parameters. Normal ranges for age: serum phosphate 1.33–2.19 mmol/L, alkaline phosphatase 145–340 U/L.

Diagnosis was made early in children born to XLH parents (mean ± SD age 0.7 ± 0.7 years) in comparison with patients with a *de novo PHEX* variant (mean ± SD age 2.1 ± 0.6 years).

All patients had a loss-of-function variant in *PHEX*, hypophosphatemia, and elevated alkaline phosphatase at diagnosis (Table 1). Auxological characteristics at baseline and during follow-up are shown in Table 2.

**Table 2:** Auxological parameters in our XLH population.

Parameters	Group 1 (burosumab)			Group 2 (phosphate supplements and vitamin D analogs)		
	*n*	Mean ± SD (SDS)	Min/max (SDS)	*n*	Mean ± SD (SDS)	Min/max (SDS)
Birth length	14	0.3 ± 0.7	−1.5/0.7	31	0.2 ± 1.0	−2.0/2.0
Birth weight	14	−0.46 ± 0.9	−2.8/0.9	31	0.1 ± 1.0	−1.9/2.0
Height at diagnosis	15	−0.7 ± 1.1	−2.5/1.9	30	−1.3 ± 1.3	−4.5/2.2
BMI at diagnosis	15	−0.7 ± 1.0	−0.8/1.9	28	0.3 ± 0.9	−1.6/2.5
Height M0	15	−1.4 ± 0.8	−2.5/0.0	30	−1.3 ± 1.1	−4.5/0.4
BMI M0	15	0.9 ± 0.9	−0.5/2.5	28	0.5 ± 1.1	−1.6/2.3
Height M6	15	−1.4 ± 0.7	−2.3/−0.2	29	−1.6 ± 1.2	−4.1/1.5
BMI M6	13	0.9 ± 0.9	−0.9/2.3	24	0.6 ± 1.1	−1.3/2.4
Height M12	15	−1.3 ± 0.7	−2.4/0.4	28	−1.8 ± 0.9	−3.7/0.1
BMI M12	15	0.7 ± 0.9	−0.9/1.9	24	0.7 ± 1.1	−1.5/2.8
Height M18	11	−1.5 ± 0.6	−2.4/−0.4	26	−1.9 ± 0.9	−3.7/0.4
BMI M18	11	0.6 ± 0.6	0.4/1.2	20	0.8 ± 1.1	−1.6/3.0
Height M24	8	−1.5 ± 0.7	−2.5/−0.6	29	−1.9 ± 1.0	−3.5/0.3
BMI M24	7	0.7 ± 0.4	0.1/1.2	28	0.9 ± 1.0	−1.0/2.9
Height at 4 years	12	−1.4 ± 0.5	−2.7/−0.6	28	−1.9 ± 0.9	−3.5/0.3
BMI at 4 years	12	0.6 ± 0.6	−0.4/1.5	28	0.8 ± 1.0	−1.2/2.9
ΔHeight M12–M0	15	0.1 ± 0.6	−0.7/1.3	28	−0.5 ± 0.8	−2.3/0.9
ΔHeight M24–M0	7	0.0 ± 0.7	−0.6/1.4	28	−0.7 ± 0.9	−2.6/1.0
ΔBMI M12–M0	13	−0.2 ± 0.7	−1.9/1.0	22	0.5 ± 0.7	−0.6/1.9
ΔBMI M24–M0	4	−0.5 ± 0.8	−1.4/0.6	25	0.5 ± 0.9	1.0/2.5

Mean ± SD, minimum, and maximum for auxological parameters. M0: initiation of therapy (burosumab for group 1, and conventional therapy for group 2), M12: after 12 months of treatment, M24: after 24 months of treatment; BMI, body mass index.

In group 1, we included 15 children, five girls and 10 boys, who started burosumab before the age of 4 years and were treated for at least 1 year. Fourteen children (93.3%) had a familial form of the disease (nine boys and five girls). The diagnosis in this group was made rather early; only three children (20%) were diagnosed after 1 year of age. Patients born to XLH parents were diagnosed at a mean age of 0.4 ± 0.7 months.

Before starting burosumab, all patients received phosphate supplements and vitamin D analogs for an average duration of 1.7 ± 0.8 years (range 3 months to 3 years).

The mean ± SD (years) age at onset of burosumab was 2.1 ± 0.7 years (range 1–2.9 years). According to the approval at the time, seven patients (46.6%) started therapy with burosumab at a dosage of 0.4 mg/kg body weight; the remaining eight patients (53.3%) began at a dosage of 0.8 mg/kg body weight. Twelve months (M12) after starting therapy, all patients had reached their maximum dose; five children (33.3%) were at the maximum dose of 2 mg/kg body weight. The mean dose of burosumab at M12 was 1.6 ± 0.5 mg/kg body weight. At the time of data collection, the mean duration of burosumab therapy was 2.7 ± 1.4 years.

In group 2, we included 31 children (19 girls, 12 boys) treated from diagnosis with phosphate supplements and active vitamin D analogs, who presented a complete dataset of both auxologic and biological parameters to be eligible for comparison. They were diagnosed later than the children in group 1. Eighteen patients (58%) were diagnosed and started treatment after 1 year of age. Twenty-four children (77.4%) had a family history of XLH (10 boys).

Patients in group 2 received oral phosphate and active vitamin D therapy in accordance with international guidelines (1) for mean ± SD duration of 7.8 ± 2.8 years (range 2.8–15.2 years). Following the study period, 26 patients (83.8%) started burosumab therapy after the age of 4 years at a mean ± SD age of 9.0 ± 3.4 years. Five patients (16%) are still receiving phosphate supplements and vitamin D analogs.

Patients in group 2 were diagnosed and started therapy significantly later than those in group 1 (*P* = 0.01 and *P* < 0.001, respectively). This difference might be the result of a faster diagnostic process for group 1, explained by advances in genetic testing.

### Burosumab and height

All the participants who received burosumab before the age of four were born full-term and eutrophic, apart from one child (6.6%) born small for gestational age (SGA). The mean ± SD birth weight SDS and length SDS were −0.46 ± 0.9 and −0.3 ± 0.7, respectively.

Following birth, height SDS declined progressively to −0.7 ± 1.1 (*P* = 0.24) at diagnosis, then to −1.4 ± 0.8 (*P* < 0.001) at the start of burosumab (mean ± SD age of 2.1 ± 0.76 years). After an average of 1 and 2 years of burosumab, the height SDS was maintained (Table 2).

We did not see any statistically significant change in Δheight SDS during the first 2 years of treatment: mean ± SD 0.1 ± 0.6 after 1 year (*P* = 0.16) and 0.0 ± 0.7 SD after 2 years (*P* = 0.54).

**Figure 2:**
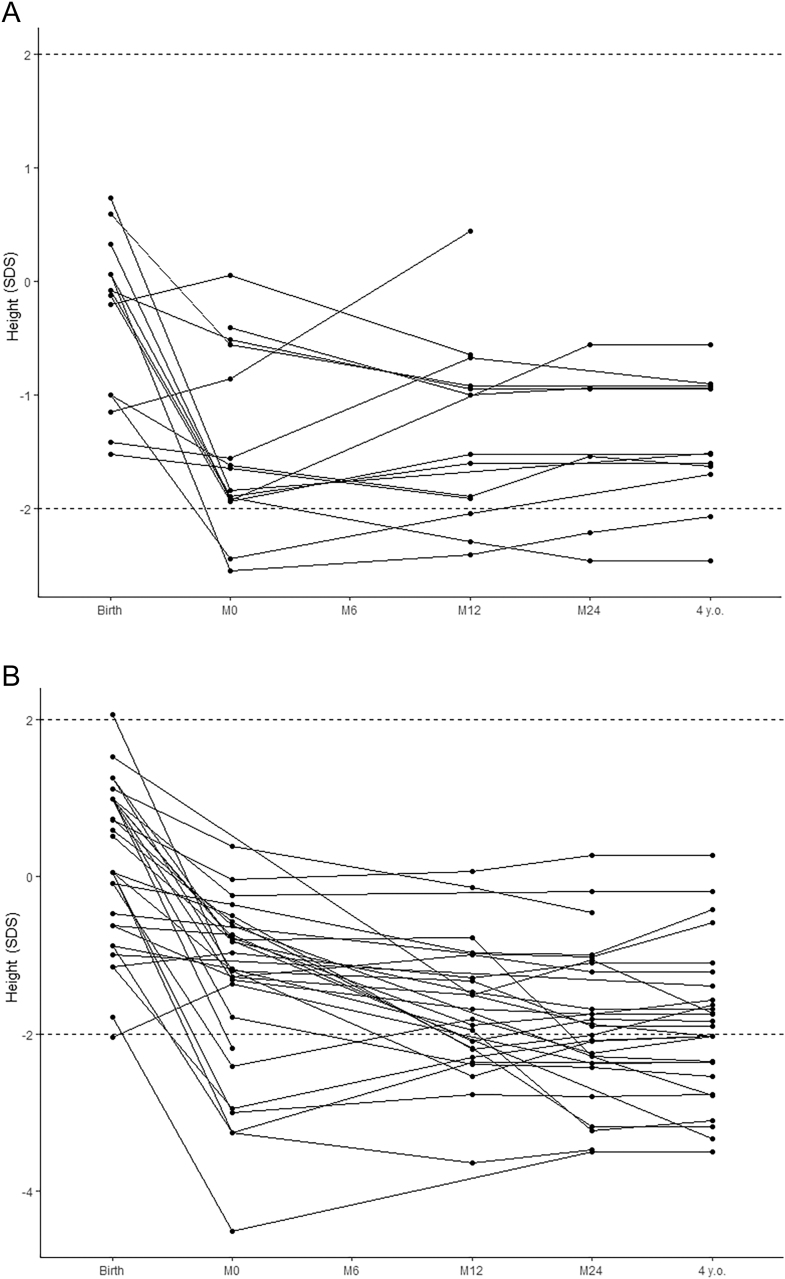
Individual growth patterns of XLH children treated before the age of four. (A) Patients who received burosumab. (B) Patients who received phosphate supplements and vitamin D analogs. The solid red lines mark +2 and −2 SDS. M0: initiation of treatment, M6: 6 months after treatment initiation, M12: 12 months of treatment, M24: 24 months of treatment.

Individual growth patterns of patients who received burosumab before the age of four years are shown in Fig. 2A.

### Oral phosphate and active vitamin D therapy and height

Except for one, children treated with oral phosphate and active vitamin D therapy were born full-term and eutrophic. In this group, we observed a continuous decline in height SDS from birth to diagnosis (−0.2 ± 0.9 SDS to −1.3 ± 1.3 SDS), through 1 and 2 years of therapy, and up to 4 years of age (−1.8 ± 0.9 SDS to −1.9 ± 0.9, respectively). The deficit in height presented at diagnosis is more important than the one seen in group 1, but not statistically significant (*P* = 0.22).

The difference in height SDS becomes even more evident when compared to birth: mean ± SD Δheight SDS of −2.0 ± 1.1, *P* < 0.001.

Individual growth patterns of patients under oral phosphate and active vitamin D therapy are shown in Fig. 2B.

**Figure 3:**
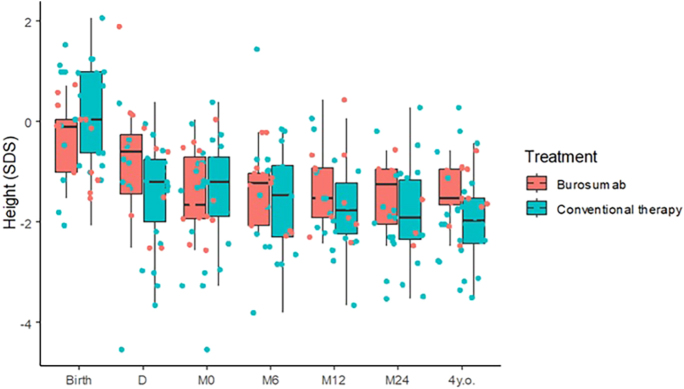
Progression and comparison of height in young children affected by XLH. Height SDSs are represented by the ‘whisker boxes’, where the median is represented by the thick black line; the 1st quartile corresponds to the bottom black line; the 2nd quartile corresponds to the top black line. D: at diagnosis, M0: initiation of therapy (burosumab for group 1 and oral phosphate and active vitamin D therapy for group 2), M6: 6 months after treatment initiation, M12: 12 months of treatment, M24: 24 months of treatment, and at the age of 4 years.

As illustrated in Fig. 3, we observed a trend toward height maintenance in patients treated with burosumab compared to those who received oral phosphate and active vitamin D therapy. There was no statistical difference between groups at any time point.

### Burosumab and BMI

During the first 2 years of treatment with burosumab, ΔBMI SDS did not change significantly, i.e., mean ± SD 0.1 ± 0.3 after 1 year (*P* = 0.54) and 0.4 ± 0.8 after 2 years (*P* = 0.37). At the age of 4 years, the mean ± SD BMI SDS was 0.6 ± 0.5. Individual progression of BMI during burosumab treatment is shown in Supplemental Fig. 1.

### Vitamin D analogs and phosphate supplements and BMI

In group 2, at the start of phosphate supplements and vitamin D analogs, the BMI SDS was within normal values (mean ± SD 0.4 ± 1.0, range −1.6–2.3). After 1 year, BMI SDS mean ± SD was 0.7 ± 1.0 (range −1.5–2.8), and after 2 years it was 0.9 ± 1.0 (range −1.0–3.0).

In comparison, we found a statistically significant variation in ΔBMI SDS in patients treated with phosphate supplements and vitamin D analogs: mean ± SD 0.5 ± 0.7 after 1 year (*P* = 0.006) and 0.5 ± 0.9 after 2 years (*P* = 0.006). At the age of 4 years, mean ± SD BMI SDS was 0.8 ± 0.97. Individual progression of BMI in this group is shown in Supplemental Fig. 2.

**Figure 4:**
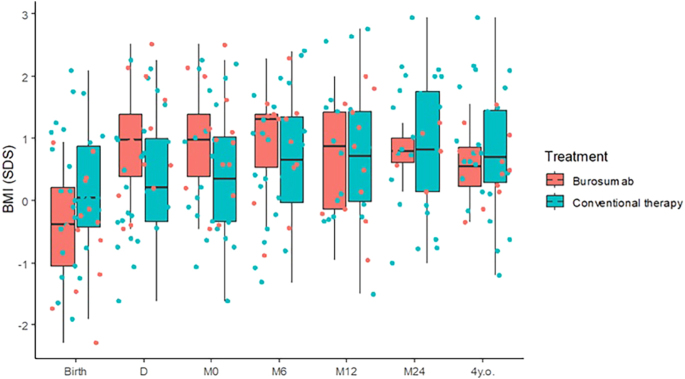
Distribution of BMI SDS in our XLH population over the follow-up period. Height SDSs are represented by the ‘whisker boxes’, where the median is represented by the thick black line; the 1st quartile corresponds to the bottom black line; the 2nd quartile corresponds to the top black line. D: at diagnosis, M0: initiation of therapy (burosumab for group 1 and oral phosphate and active vitamin D therapy for group 2), M6: 6 months after treatment initiation, M12: 12 months of treatment, M24: 24 months of treatment, and at the age of 4 years.

The progression of BMI SDS over time in XLH children is shown in Fig. 4. Despite a significant increase of BMI in patients treated with oral phosphate and active vitamin D therapy, we did not observe statistical differences at any time points between the groups.

### Progression of rickets

In patients treated with phosphate supplements and vitamin D analogs or with burosumab, we observed a significant decrease in ALP levels from 689 ± 469 IU/L to 305 ± 106 IU/L and from 533 ± 313 IU/L to 349 ± 51 IU/L, respectively, between diagnosis and 4 years of age.

**Figure 5:**
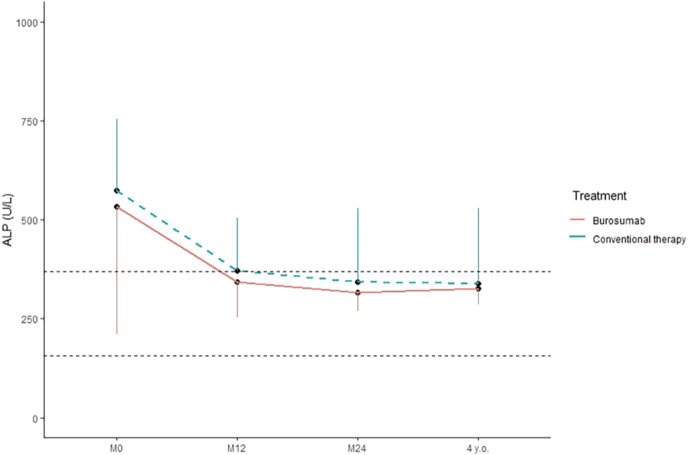
Alkaline phosphatase levels in our XLH population over the follow-up period. Dashed black lines mark the upper and lower limits of the normal interval (50–390 IU/L).

There was no significant difference in the progression of ALP levels between oral phosphate and active vitamin D-treated participants and burosumab-treated children, as seen in Fig. 5. We correlated plasma ALP values (IU/L) with height (SDS) at the various time points in the two groups.

In the burosumab group, we found no correlation between height SDS and ALP values at M0 (*P* = 0.2), M24 (*P* = 0.9) or 4 years of age (*P* = 0.7). In contrast, at M12, the correlation was statistically significant (*P* = 0.03).

In the group treated with phosphate supplements and vitamin D analogs, we found no correlation between height SDS and ALP values at any time point: *P* = 0.3, *P* = 0.9, *P* = 0.2 and *P* = 0.3, respectively, at M0, M12, M24, and 4 years of age.

The changes in biochemical parameters during treatment are shown, in a descriptive manner, in the Supplemental Table. As expected, children treated with burosumab showed an increased plasma phosphate level, which remained stable over time.

## Discussion

We report, for the first time, the effect of burosumab on growth in a large cohort of very young children affected by XLH. In addition, we compared the progression of height, BMI, and biochemistry of young children treated with burosumab to that of young children treated with phosphate supplements and vitamin D analogs.

Since vitamin D analogs are available, growth in XLH children has been followed as a marker of rickets and disease severity (8, 18, 19). Clinical studies have shown a controversial effect of supplementation therapy by phosphate and vitamin D analogs on linear growth. Some studies have reported an improvement in final height, while others have shown no significant effect on growth (8, 20, 21). Overall, the combination of phosphate supplements and vitamin D analogs seems to have only a minor impact, as many individuals reach a final height below −2 SDS (11). Very few data are available on growth during infancy and early childhood. Reports suggest an improvement in height when phosphate supplements and vitamin D analogs are started before the onset of rickets symptoms (22, 23). In addition, an early start of treatment is associated with better outcomes (19).

Studies analyzing the effects of burosumab on linear growth seem to be disputed. Carpenter *et al.* documented a significant improvement of mean standing-height SDS in children aged five to 12 years (9); this increase is, however, minimal, (i.e., height gain SDS of 0.19 ± 0.05 in children treated with burosumab). In contrast, Whyte *et al.*, in a phase 3 study, found no significant improvement in young children (aged 1–4 years) (24).

Ward *et al.* showed that in children aged 1 to 5 years, treated with burosumab, linear growth improved modestly from baseline to week 64 (mean recumbent length or standing height Z-score gain of 0.15), with the greatest growth during the first 6 months (13).

Our study analyzed the early growth of 48 children. We established that XLH is associated with a decline in height, which occurs during the first 2 years after birth. This supports the initiation of treatment from diagnosis and, if possible, from birth.

We also showed that burosumab prevents further height loss in XLH children, whereas oral phosphate and active vitamin D therapy is associated with continuous height decline.

Our data indicate that although burosumab improves plasma phosphate levels, it does not correct the height deficit. Probably, in XLH, phosphate-independent mechanisms involving excess FGF23 or directly the *PHEX* gene play a key role in the growth deficit; these mechanisms have yet to be identified.

However, although not statistically significant, burosumab, in comparison with oral phosphate and active vitamin D therapy, would appear to prevent the loss of centimeters, especially in the first year after treatment initiation. This effect could be explained by a better correction of rickets and, consequently, a reduction in lower extremity deformities during burosumab treatment. If this is the case, it would be desirable to start burosumab therapy before the onset of lower limb deformities in order to avoid or limit the initial growth deficit.

It should also be noted that patients on oral phosphate and active vitamin D therapy, unlike those treated with burosumab, already have a significant stature deficit at diagnosis. This difference between the two groups could have multiple explanations: i) the patients are older at diagnosis in the oral phosphate and active vitamin D therapy group (mean ± SD age at diagnosis 1.2 ± 0.8 years in group 2 vs mean ± SD age at diagnosis 0.5 ± 0.8 months in group 1); ii) children in group 2 were treatment-naïve before initiating oral phosphate and active vitamin D therapy, whereas, all the children in group 1 were on oral phosphate and active vitamin D therapy before starting burosumab, and this may have partially limited the growth deficit compared to untreated patients.

There are no literature data on BMI or weight progression in children treated with burosumab at an early age. In a previous study, we found comparable BMI SDS after 1 year of burosumab in prepubertal children (16). According to these results, in our study, BMI in burosumab-treated patients did not change during the first 2 years. In contrast, in patients on oral phosphate and active vitamin D therapy, we noted a small but statistically significant increase in BMI SDS during the first 2 years of therapy. These findings are in agreement with other studies, in which hypophosphatemia would seem to correlate with weight gain and metabolic syndrome (25). Through the improvement of phosphate levels, burosumab prevents the increase of BMI and related long-term complications (hypertension, obesity, and glucose intolerance), often seen as complications in XLH. Moreover, this effect could also be explained by an increase in physical activity and mobility of patients, secondary to the better control of rickets and associated symptoms during burosumab therapy. In order to confirm this positive metabolic effect, long-term studies are needed.

As seen in the literature, both therapies improve the control of rickets, demonstrated by the normalization of alkaline phosphatase values after the first year of therapy (26, 27, 28, 29). Imel *et al.* found in 61 XLH patients aged between one and 12 years a statistically significantly better reduction in plasma alkaline phosphatase in the group of patients on burosumab when compared to those on oral phosphate and active vitamin D therapy, after 16 weeks of therapy (mean ± SD −18 ± 11% in the burosumab group vs 0 ± 21%, *P* < 0.0001) (12). Our study shows that early treatment is more effective than therapy started later in controlling rickets markers. The absence of a significant difference in ALP values in our sample could be explained by the fact that, in accordance with French guidelines, early therapy with burosumab was started in the most severe clinical forms, which therefore had greater cartilage damage, or in patients in whom the administration of oral phosphate and active vitamin D therapy was not tolerated, and therefore rickets was still in an active phase.

In addition, to test whether better control of rickets (ALP within normal limits) was related to a better stature outcome, we correlated alkaline phosphatase values with height SDS at different time points. We did not find any statistically significant correlation in patients treated with burosumab or in patients treated with oral phosphate and active vitamin D therapy. The only exception was the statistically significant correlation at M12 in the burosumab group. This result could explain the stable growth rate in the first year of therapy: better control of rickets allows better linear growth, by both correcting bone deformities and maintaining more stable phosphate levels.

Our study has several limitations. Adherence to phosphate and vitamin D supplementation was not measured by other means than the ALP levels. As acknowledged in several studies, due to multiple daily administration and the possibility of gastrointestinal side effects, therapeutic adherence is often suboptimal, resulting in bias in the interpretation of results. Moreover, oral phosphate and active vitamin D therapy administration, before switching to burosumab, might have biased the statistical comparison between the two groups. In reality, this bias cannot be avoided, as burosumab treatment is recommended after the age of one.

## Conclusion

Our study is the first to present data on growth patterns in very young XLH children treated with burosumab. We found that after 2 years of treatment, burosumab prevented further loss of centimeters, which occurs in patients on oral phosphate and active vitamin D therapy. We believe that it would be advantageous to start burosumab therapy as early as possible.

In addition, burosumab is associated with a positive metabolic effect, which prevents, rather early, the increase in weight and, consequently, the long-term effects of metabolic syndrome.

In conclusion, we consider it important to start burosumab as soon as possible in order to limit the early damaging impact of XLH on cartilage and on linear growth, and to avoid further metabolic complications.

Nowadays, we plan for early/neonatal screening of XLH. It is therefore very important to know that features of XLH develop very early in life and can be prevented by appropriate therapy.

## Supplementary materials





## Declaration of interest

DAE reports receiving travel grants and honoraria from Novo Nordisk, Pfizer, Kyowa Kirin, and Merck Serono. AL reports receiving a research grant, travel grants, and honoraria from Novo Nordisk, Pfizer, Alexion, Kyowa Kirin, Sandoz, and Merck Serono. All disclosures mentioned are unrelated to this research project. The other authors have no conflicts of interest.

## Funding

This work was partially supported by the 2023 Early Career Scientific Development Grant from the European Society for Paediatric Endocrinology.

## Author contribution statement

DAE and AL designed the study. ES, DAE, AR, ASL, and AL collected the data. JB, ES, and DAE analyzed the data. DAE, ES, and AL interpreted the data. DAE, ES, and AL drafted the manuscript. All authors critically reviewed the manuscript and provided significant input. All authors read and approved the final version of the manuscript. DAE and AL take responsibility for the integrity of the data and its analysis.

## Data availability

The data that support the findings of this study are available from the corresponding author upon reasonable request.

## Ethics

The study was approved by the French Data Protection Authority (CNIL), under the promotion of INSERM (Institut National de la Sante et de la Recherche Médicale). Consent has been obtained from each patient and subject after full explanation of the purpose and nature of all procedures used.
