# Supplementary material for: Burosumab prevents further height deficit in toddlers affected by XLH
Source: Endocr Connect. 2025 Oct 18;14(10):e250435. doi: 10.1530/EC-25-0435 (PMC12538273; doi:10.1530/EC-25-0435)
Supplement: Supplementary file 1 [file supplementary_figures.pdf]

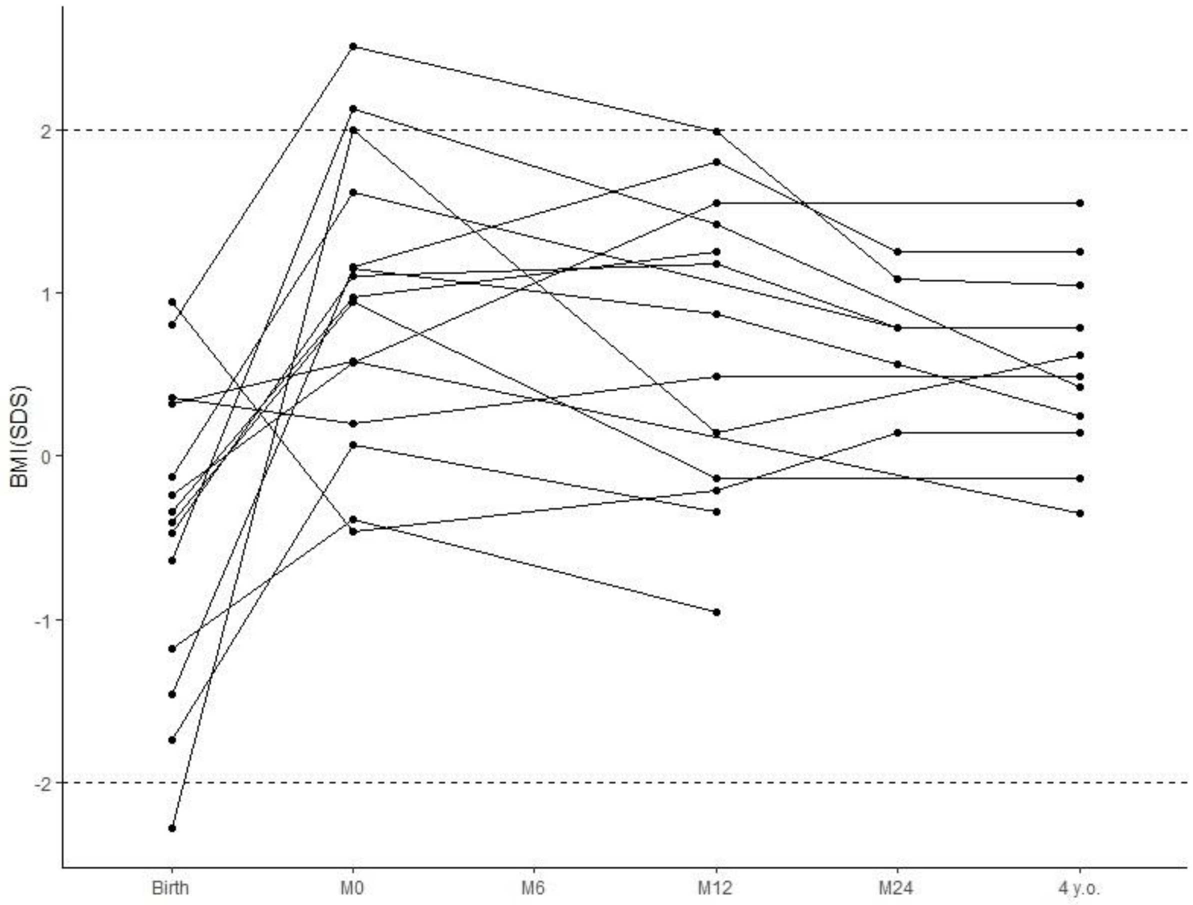

[Figure 1] Individual changes in BMI SDS in patients who received burosumab before the age of four years. The solid red lines mark +2 and − 2 SDS. M0: initiation of burosumab, M6: 6 months after treatment initiation M12: 12 months of treatment, M24: 24 months of treatment.

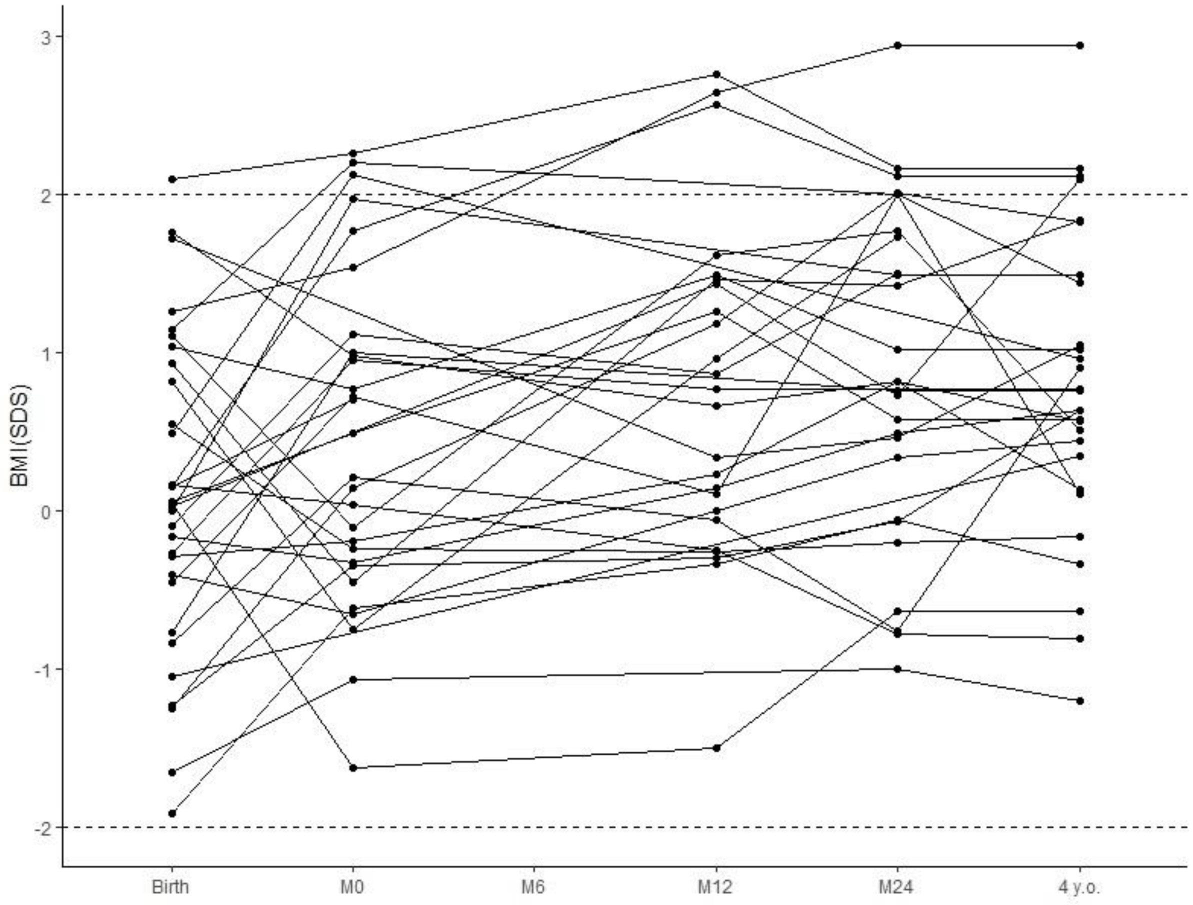

[Figure 2] Individual changes in BMI SDS in patients who received phosphate supplements and vitamin D analogs before the age of four years. The solid red lines mark +2 and − 2 SDS. M0: initiation of oral phosphate and active vitamin D therapy, M6: 6 months after treatment initiation M12: 12 months of treatment, M24: 24 months of treatment.
