# Supplementary material for: Burosumab prevents further height deficit in toddlers affected by XLH
Source: Endocr Connect. 2025 Oct 18;14(10):e250435. doi: 10.1530/EC-25-0435 (PMC12538273; doi:10.1530/EC-25-0435)
Supplement: Supplementary file 2 [file supplementary_table.pdf]

Supplemental table: Biochemical changes in the study population

Parameter	Group 1 (burosumab)				Group 2 (phosphate supplements and vitamin D analogs)		
	**Diagnosis**	**M0**	**M12**	**M24**	**M0**	**M12**	**M24**
**Phosphate (mmol/L)**	1.14 ± 0.25	0.84 ± 0.16	1.16 ± 0.18	1.04 ± 0.2	0.84 ± 0.22	1.22 ± 0.38	0.93 ± 0.12
**ALP (IU/L)**	744 ± 226	732.9 ± 323.6	358 ± 76.7	316 ± 43.1	885.3 ± 617	492 ± 119.1	296.7 ± 105.7
**PTH (ng/L)**	57.9 ± 29.4	42.1 ± 26.6	43.8 ± 20.8	57.7 ± 23.0	55.3 ± 29.1	21.7 ± 9.71	21.4 ± 3.3
**Calcium (mmol/L)**	2.5 ± 0.08	2.46 ± 0.13	2.45 ± 0.11	2.37 ± 0.13	2.45 ± 0.09	2.39 ± 0.10	2.50 ± 0.11

Supplemental table: Biochemical changes in the study population

**25OH vitamin D (ng/mL)**	47.3 ± 19.2	34.7 ± 12.5	36.1 ± 12.6	37.4 ± 14.9	37.6 ± 15.6	36.5 ± 4.95	42.0 ± 19.5
**TRP %**	85 ± 12.7	79.1 ± 12	88.7 ± 5.4	88.4 ± 3.8	85.1 ± 9.6	data not available	64.2 ± 6.5

Mean ± SD are shown. Normal ranges for age: serum phosphate 1.33-2.19 mmol/L, alkaline phosphatase 145-340 U/L, PTH 12-88 ng/L, serum calcium 2.20-2.70 mmol/L, 25-hydroxyvitamin D 30-100 ng/mL.
